# Lung Cavitation as a Long-Term Imaging Pattern of COVID-19

**DOI:** 10.7759/cureus.39825

**Published:** 2023-06-01

**Authors:** Caner Çınar, Derya Kocakaya, Sehnaz Olgun Yıldızeli, Sait Karakurt

**Affiliations:** 1 Pulmonology Department, Marmara University, Istanbul, TUR; 2 Pulmonology Department, Marmara University School of Medicine, Istanbul, TUR

**Keywords:** long covid-19, post-covid-19, computerized tomography, lung cavitation, covid-19

## Abstract

Background

A wide variety of radiological imaging findings, especially CT findings, have been reported in patients with COVID-19 pneumonia during the pandemic surge. Generally, on control chest imaging, individuals who have been cured of the disease usually show complete remission; however, in severe cases, residual pulmonary fibrosis, other abnormalities, and, rarely, lung cavitation can be observed. In this retrospective descriptive study, we aimed to describe the clinical, radiological, and laboratory characteristics of patients who developed lung cavitation in the course of SARS-CoV-2 disease recovery.

Methodology

Over a period of five months from March 1, 2021, to August 1, 2021, 15 consecutive patients who developed cavitary lesions on chest CT during the course of recovery from COVID-19 were recruited as the study population. All patients had a history of a SARS-CoV-2 infection diagnosed with a positive real-time polymerase chain reaction test. Patients who already had cavitary lesions in chest CT during the start of COVID-19 symptoms were excluded.

Results

In this study, 14 patients were male (93.3%). The only female patient was the only severely obese patient in the study population, with a body mass index was 40.4 kg/m^2^. The median (range) age of the patient population was 61 (42-79) years. Eight patients (53.3%) required intensive care unit admission during the hospitalization period. Three patients who required intensive care unit were intubated and needed invasive mechanical ventilation. Two patients died during hospitalization.

Conclusions

Lung cavitation remains a rare occurrence in the course of COVID-19. Bronchoscopic evaluation and scanning for pulmonary embolism should be done in appropriate patients to determine secondary reasons for cavitation. Although this descriptive study showed that cavitary lesions can develop in patients with severe disease, more comprehensive studies with a control group are needed to reach a definitive conclusion.

## Introduction

During the last months of 2019, the SARS-CoV-2 outbreak began in Wuhan, China. On March 2020, the WHO declared COVID-19 a global pandemic [1]. The highly contagious pneumonia caused by the COVID-19 pandemic spread quickly around the world and resulted in millions of deaths. Mild COVID-19 is characterized by fever, malaise, cough, upper respiratory symptoms, and/or less common features of COVID-19, in the absence of dyspnea. The majority of patients do not need to be hospitalized. Patients generally require hospitalization if they experience dyspnea, which raises concerns that they have a condition of at least moderate severity. Patients may have infiltrates on chest imaging and still be thought to have a moderate illness, but the presence of any of the following signs or symptoms suggests severe disease: hypoxemia (oxygen saturation 94% on room air), the requirement for oxygenation, or ventilatory support [2].

In cases of moderate or early illness, chest radiographs may be normal. In a retrospective analysis of 64 COVID-19 patients in Hong Kong, 20% never had any abnormal chest radiograph findings at any stage in the disease [3]. Consolidation and ground-glass opacities with bilateral, peripheral, and lower lung zone distributions were frequent abnormal radiographic findings. Lung involvement developed throughout the course of the illness, reaching a peak in severity 10 to 12 days following the onset of symptoms.

A wide variety of radiological imaging findings, especially chest CT findings, have been reported in patients with COVID-19 pneumonia. Although some chest CT abnormalities may be indicative of COVID-19 and chest CT may be more sensitive than a chest radiograph, no CT finding can definitively confirm or deny the presence of COVID-19. Chest CT should only be used for hospitalized patients when needed for treatments, according to the Society of Thoracic Radiology, the American College of Radiology, and the Radiological Society of North America which warn against using CT for COVID-19 screening or diagnosis [4].

Most frequently, ground-glass opacification with or without consolidative abnormalities is seen on chest CT in COVID-19 patients, which is consistent with viral pneumonia. On control chest imaging, individuals who have been cured of the disease usually show complete remission; however, in severe cases, residual pulmonary fibrosis and other abnormalities can be observed [5]. Long-term imaging or sequelae pattern of COVID-19 is not well defined yet. In this study, our aim was to describe the clinical, radiological, and laboratory characteristics of 15 patients admitted to Marmara University Hospital between March 1, 2021, and August 1, 2021, who developed cavitary lesions in the course of SARS-CoV-2 disease recovery.

## Materials and methods

Patients

The study was conducted at Marmara University, School of Medicine Pulmonary and Critical Care Medicine Clinic. Ethical approval was obtained from the Marmara University, School of Medicine Clinical Research Ethics Committee (approval number: 5.11.2021;1224).

In this retrospective and descriptive study conducted over a period of five months from March 1, 2021, to August 1, 2021, 15 consecutive patients who developed cavitary lesions on chest CT during the course of recovery from COVID-19 were recruited as the study population. Patients who already had cavitary lesions on chest CT during the start of COVID-19 symptoms were excluded.

Demographics and comorbidities

Gender, age, concomitant conditions, body mass index (BMI), smoking status, and length of hospital stay were documented for all patients.

Standard treatment

Treatment planning for COVID-19 was implemented following the guidelines prepared by the Republic of Turkey, Ministry of Health [6]. Treatment modifications were made according to the patients’ daily physical examinations and laboratory findings by pulmonary medicine and critical care physicians during the hospital stay.

The recommended treatment scheme according to the guidelines of the Ministry of Health is favipiravir 600 mg bid after 1,600 mg bid loading dose in patients for five days, which in severe patients can be extended up to 10 days. Low-molecular-weight heparin was given to all hospitalized patients as an anticoagulant. Patients with high oxygen demand, severe respiratory distress, and who had documented embolism were treated with enoxaparin 0.02 mg/kg/day bid per day, and clinically stable patients were treated with enoxaparin 0.01 mg/kg/day once daily.

Patients who had mild hypoxemia were treated with 6 mg dexamethasone or an equivalent corticosteroid dose. For patients with higher oxygen demand, needing mechanical ventilation, and those with evolving cytokine release storm (CRS), higher doses of corticosteroids were added to the treatment regimen, and the dose was tapered according to clinical response during the hospital stay.

Empiric antibacterial or antifungal treatment was administered only if there was clinical suspicion of secondary bacterial or fungal pneumonia. For patients with evolving CRS during disease surge, anti-tumor necrosis factor (TNF) tocilizumab was recommended. Initially, one dose was given, and a second dose was optional based on the clinical evolution of the patient.

Laboratory parameters

In all cases; lymphocyte count (×10³/μL), platelet count (×10³/µL), mean platelet volumes, neutrophil/lymphocyte count, lymphocyte/C-reactive protein (CRP) and platelet/CRP ratios, D-dimer (mg/L), CRP (mg/L), procalcitonin (μg/L), ferritin (μg/L), and lactate dehydrogenase (LDH) (U/L) findings at admission were recorded.

Disease severity

Clinical staging for all patients during hospitalization was calculated based on the values of the worst clinical findings during hospitalization according to the WHO progression scale [7]. Radiologic severity was assessed using CT visual quantitative evaluation from 0-20 [8]. Each lung lobe was assessed for the percentage of lobar involvement and classified as none (0%), minimal (1-25%), mild (26-50%), moderate (51-75%), or severe (76-100%), with corresponding scores of 0, 1, 2, 3, or 4. The total severity score was reached by summing the five lobe scores. All radiological scorings were done by a single expert. Patients clinically suspected of having pulmonary embolism or venous thromboembolism were evaluated with deep venous ultrasonography or pulmonary CT angiography, and those suspected to have secondary infections were evaluated for microbiological pathogens with the aid of sputum culture, blood culture, or bronchoscopic evaluation.

Statistical analysis

Data obtained in the study were analyzed using SPSS Statistics for Windows version 23.0 software (IBM Corp., Armonk, NY, USA). Continuous variables were stated as mean ± standard deviation (SD) and categorical data as numbers (n) and percentages (%).

## Results

**Figure 1:**
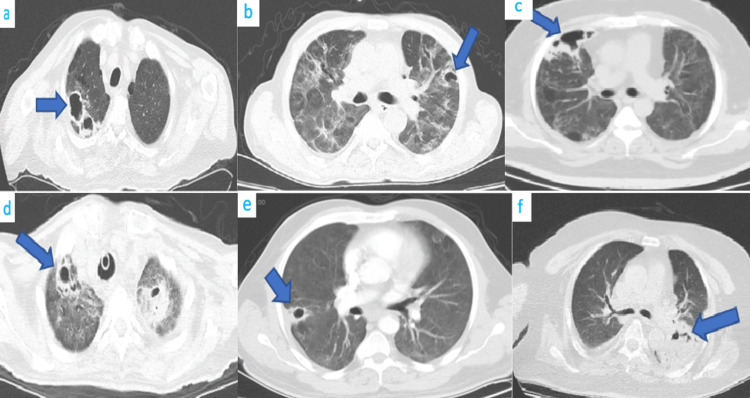
CT images of six patients. Cavitary lesions are located differently in patient groups. In a and d, cavitations are located at apices. In b, c, d, and f, cavitary lesions are located at the lower lobes.

A total of 1,382 patients having COVID-19 were admitted to Marmara University Hospital Pulmonary Medicine and Critical Care clinic during the study period. Of the 1,382 patients, 15 had developed cavitary lesions during the disease course. CT images of six patients are demonstrated in Figure 1.

Demographic features, treatments, length of hospital stay, and laboratory findings of the study population on admission are summarized in Table 1 and Table 2.

**Table 1:** Summary of demographic features, length of hospital stay, and treatments. F: female; M: male; HT: hypertension; GERD: gastroesophageal reflux disease; TNF: tumor necrosis factor; ARB: acid-resistant bacteria; COPD: chronic obstructive pulmonary disease; CAD: coronary artery disease; CVE: cerebrovascular event *: Steroid doses converted to dexamethasone and total dose taken divided by hospitalization time when mean dexamethasone dose was calculated.

	Patient 1	Patient 2	Patient 3	Patient 4	Patient 5	Patient 6	Patient 7	Patient 8	Patient 9	Patient 10	Patient 11	Patient 12	Patient 13	Patient 14	Patient 15
Age (years)	64	68	42	62	52	61	56	70	47	61	45	42	59	79	65
Sex	F	M	M	M	M	M	M	M	M	M	M	M	M	M	M
Comorbidities	Obesity, asthma	-	HT	-	Chronic hepatitis B	GERD	-	CAD, COPD, ischemic CVE	HT, Behçet’s disease	Peptic ulcers	-	Wegener	-	COPD	-
BMI (kg/m^2^)	40.4	24.5	26.5	25	23.1	26.8	25.7	29.4	26.3	28.1	27.4	31.3	26.8	28.5	27.8
Smoking history	-	30 pack-years	-	-	-	30 pack-years	-	40 pack-years	20 pack-years	12 pack-years	15 packs-years	10 pack-years	15 pack-years	30 pack-years	40 pack-years
Length of hospital stay (days)	45	20	55	93	48	35	22	26	34	48	70	60	30	10	15
Mean dexamethasone dose(mg/day)*	16.3 mg/day	4.1 mg/day	9.78 mg/day	5.7 mg/day	7.37 mg/day	6 mg/day	19 mg/day	6 mg/day	6.4 mg/day	15.6 mg/day	10.2 mg/day	12.4 mg/day	11.3 mg/day	11.4 mg/day	8.4 mg/day
Highest dexamethasone dose taken in one day (mg)	40 mg	8 mg	20 mg	20 mg	20 mg	20 mg	3 × 250 mg methylprednisolone (mini-pulse)	10 mg	40 mg	3 × 250 mg methylprednisolone (mini-pulse)	3 × 250 mg methylprednisolone (mini-pulse)	3 × 250 mg methyl prednisolone (mini-pulse)	20 mg	20 mg	40 mg
Favipiravir use	5 days	5 days	5 days	5 days	5 days	5 days	10 days	5 days	5 days	5 days	5 days	5 days	5 days	5 days	5 days
Anti TNF (tocilizumab use)	-	-	2 doses 400 mg	1 dose 400 mg	-	-	1 dose 400 mg	-	-	-	1 dose 400 mg	-	-	-	-

**Table 2:** Laboratory findings of the patients on admission. WBC: white blood cell; MPV: mean platelet volume; LDH: lactate dehydrogenase; CRP: C-reactive protein

	Patient 1	Patient 2	Patient 3	Patient 4	Patient 5	Patient 6	Patient 7	Patient 8	Patient 9	Patient 10	Patient 11	Patient 12	Patient 13	Patient 14	Patient15
Lymphocytes (1.5–3.1 × 10³/µL)	0.7	0.7	1.5	0.6	0.6	4.7	0.4	0.7	1.1	0.3	1.4	0.8	0.5	0.3	1.4
Neutrophil/Lymphocyte ratio	18.28	8.57	2.8	26.5	14.5	1.08	36.25	4.57	6.71	62.6	6.71	6.62	18.4	9.6	12.6
Lymphocyte/CRP ratio	60.8	8.35	17.54	2.68	8.91	758	10.6	15.6	95.4	6.52	9.45	8.55	3.2	4.68	18.18
Platelet/Lymphocyte ratio	0.016	0.47	0.172	0.63	0.173	0.05	0.775	0.22	0.15	1.01	0.06	0.27	0.31	0.91	0.16
MPV	10.1	11.1	11.2	12.1	9.3	10	10.3	10.9	10.7	12.3	12.7	11.6	11.7	9.7	11.2
Platelets (150–440 × 10³ /µL)	388	333	259	380	104	267	310	154	318	305	96	219	157	273	225
D-dimer (0–0.5 mg/L)	0.27	5.19	0.33	16.12	2.68	0.7	8.45	3.24	8.56	0.43	2.23	0.69	4.88	2.09	1.28
LDH (0–250 IU/L)	364	865	538	559	437	304	302	404	401	313	847	519	900	420	325
Ferritin (30–400 µg/L)	50.8	1647	702	946	227.8	463	773.2	386	874	100.8	1354	1204	2173	642	850
CRP (0–5 mgd/L)	11.5	83.8	85.5	223.5	67.3	6.2	37.7	44.8	22	46	148	93.5	156	64	77
Procalcitonin (0–0.5 µg/L)	0.03	0.11	0.2	0.63	1.38	0.06	0.11	0.06	0.09	0.12	0.13	0.86	2.37	0.05	0.2

In total, 14 (93.3%) patients were male. The only female patient was severely obese, with a BMI of 40.4 kg/m^2^. The median (range) age of the patients was 61 (42-79) years. Ten of the 15 patients had a smoking history. Two patients had been diagnosed with chronic obstructive pulmonary disease and were using inhalers. The mean length of hospital stay was 40.7 days, ranging between 10 and 93 days. Eight (53.3%) patients required intensive care unit admission during the hospitalization period. Three of the patients who required intensive care unit admission were intubated and needed invasive mechanical ventilation. Two patients died during hospitalization. Thirteen patients were discharged after treatment.

All patients were treated with the antiviral drug favipiravir for at least five days and corticosteroids, with the dose adjusted and tapered according to clinical response. Dexamethasone dose ranged between 4.1 mg/day and 19 mg/day during hospitalization. The highest total dose taken by patients in one day is also shown in Table 1. Four (26.6%) had CRS, and tocilizumab was added to their regimen.

In total, 13 (86%) patients had lymphopenia, 13 (86%) had elevated D-dimer titers, 11 (73.6%) had elevated ferritin levels, and all had increased LDH on admission. The neutrophil/lymphocyte ratio was above 3 in 13 (86%) patients. Lymphocyte/CRP and platelet/CRP ratios and other laboratory values on admission are also shown in Table 2.

Visual quantitative evaluation scores of the study population were between 6 and 20. The mean cavitary lesion detection time after SARS-CoV-2 PCR positivity was 53.2 days (range = 23-116 days). Six (40%) patients had documented pulmonary embolism during the follow-up period. Galactomannan was positive in two patients. Two patients had *Mycobacterium tuberculosis* complex positivity, and five patients had grown bacterial pathogens in microbiological cultures. The longest diameter of cavitary lesions ranged between 9 and 52 mm. Cavitary lesions were located bilaterally in three patients. Other characteristics of cavitary lesions are summarized in Table 3.

**Table 3:** Location of the cavitary lesions, microbiological culture results, documented VTE presence, and WHO clinical progression scale of patients. DVT: deep vein thrombosis; VTE: venous thromboembolism; MTB: *Mycobacterium tuberculosis*; BAL: bronchoalveolar lavage; DTA: deep tracheal aspirate; MSSA: methicillin-sensitive *Staphylococcus aureus*; MRSA: methicillin-resistant *Staphylococcus aureus*; sPE*: segmental pulmonary embolism; **: BAL cultures; ^^: sputum culture; RUL: right upper lobe; LUL: left upper lobe; RML: right middle lobe; RLL: right lower lobe; LLL: left lower lobe

	WHO clinical progression scale	Radiological score	Documented VTE	Culture results	Cavitary lesion detection time after PCR positivity (days)	Location of cavitary lesions	Number of cavities	Wall thickness of cavitary lesions	Longest diameter of cavitary lesions
Patient 1	5	6	Bilateral sPE*	MTB complex**	42	RLL	2	4 mm	13 mm
Patient 2	5	9	Left femoral vein DVT+bilateral sPE*	-**	23	LLL	2	5.6 mm	12 mm
Patient 3	6	12	-	Galactomannan+**	52	RUL	2	8 mm	39 mm
Patient 4	9	20	-	K. pneumonia**	56	RUL+LUL	2	3.1 mm	18 mm
Patient 5	6	15	Bilateral sPE*	MTB complex**	58	LLL	2	3.9 mm	16 mm
Patient 6	6	19	Bilateral sPE*	-**	61	RUL	1	3.4 mm	14 mm
Patient 7	10	19	-	MSSA^^	23	RML	1	5.14 mm	24 mm
Patient 8	5	20	-	-**	116	RUL	2	5.8 mm	39 mm
Patient 9	6	20	Bilateral sPE*	K. pneumoniae**	70	LLL	1	1.58 mm	47 mm
Patient 10	6	12	-	K. pneumoniae**	50	LUL	1	5.4 mm	52 mm
Patient 11	6	13	Bilateral sPE*	-^^	34	RLL+LLL	3	3.7 mm	49 mm
Patient 12	8	9	-	MRSA^^	26	LLL	1	3.2 mm	44 mm
Patient 13	5	9	-	-	82	RUL	1	7 mm	11 mm
Patient 14	5	7	-	-	32	RLL	1	6 mm	9 mm
Patient 15	5	12	-	Galactomannan+**	74	RUL+LUL	2	5.1 mm	37 mm

## Discussion

Peripheral, bilateral, diffuse ground-glass opacities or multifocal ground-glass opacities of rounded morphology with or without consolidation, as well as halo and inverted halo signs, are common CT findings of COVID-19 in the acute period [9]. During the healing stages of the disease, the lung lesions can gradually disappear and rarely fibrosis can be seen. However, long-term imaging patterns are still not known requiring further observations [10]. Cavitary lung lesions are classically known to be related to fungal, mycobacterial, autoimmune, parasitic, or neoplastic etiologies. Increased use of steroids and anti-TNF drugs in the treatment of SARS-CoV-2 patients can also be the cause of cavitation due to secondary infections such as *Mycobacterium tuberculosis* complex detected in two of our cases. Patients who developed cavitation during follow-up should also be checked carefully for fungal infections and secondary bacterial infections. Five of our patients had bacterial growth which could lead to cavitary lesions (*Klebsiella pneumoniae*, MSSA, MRSA) and two were galactomannan positive in bronchoalveolar lavage fluid. Pulmonary embolism is a known cause of infarction and lung cavitation, and SARS-CoV-2 patients have a higher tendency toward coagulation [11]. Six (40%) of our patients had documented pulmonary embolism.

However, in some patients, no underlying condition is found to cause cavitary lesions on chest CT, and the only reason may be the COVID-19 virus itself. Although the specific process of cavitation in COVID-19 patients is unknown, autopsy reports have suggested that it may arise following diffuse alveolar injury, intra-alveolar hemorrhage, and parenchymal cell necrosis [12]. Patients with more severe disease can have a higher tendency to develop cavitary lesions. All patients in our study population were hypoxemic and hospitalized. Their worst WHO clinical progression scores ranged between 5 and 10 during hospitalization. Thirteen patients have increased neutrophil/lymphocyte ratio and the same patients had lymphopenia on admission. Liu et al. found a relationship between poor prognosis in COVID-19 patients and increased neutrophil/lymphocyte ratio [13]. Another study showed lymphopenia at the first presentation of COVID-19 to be associated with disease severity [14].

Compared to women, men with COVID-19 experience more severe disease and higher mortality. The male sex is an independent risk factor for developing more severe COVID-19 disease [15]. Fourteen (93.3) patients were male in our study population, whereas the only female patient was severely obese (BMI >40 kg/m^2^). Dose-response relationship between higher BMI and severe COVID-19-associated illness has been highlighted in previous reports, with obesity being another risk factor for severe infection [16]. These demographic characteristics and laboratory values support cavitary lesions developing in patients with more severe COVID-19 in our study.

Our study had some limitations. This is a single-centered, descriptive, retrospective analysis of COVID-19 patients. Every patient with cavitation did not have a chance to undergo an investigation for pulmonary embolism accurately or bronchoalveolar lavage because of their clinical instability and high oxygen demand. Because only patients who underwent chest CT were screened retrospectively, patients who developed similar cavitary lesions during the study period would have been missed.

## Conclusions

Lung cavitation remains a rare occurrence in the disease course of COVID-19 patients. Every patient who developed a cavitary lesion during the post-COVID-19 follow-up must be evaluated individually according to their symptoms and findings. Bronchoscopic evaluation and scanning for pulmonary embolism should be done in appropriate patients to determine secondary reasons for cavitation. This descriptive study showed that cavitary lesions can develop in patients with severe disease, but more comprehensive studies with a control group are needed to reach a definitive conclusion. The need for prophylaxis (e.g., antifungal, antituberculosis) before administering long-term steroids or anti-TNF drugs could also be a future topic of discussion in COVID-19 patients.
